# Key Metabolic Pathways in MSC-Mediated Immunomodulation: Implications for the Prophylaxis and Treatment of Graft Versus Host Disease

**DOI:** 10.3389/fimmu.2020.609277

**Published:** 2020-12-07

**Authors:** Andre J. Burnham, Elisabetta Manuela Foppiani, Edwin M. Horwitz

**Affiliations:** ^1^ Aflac Cancer & Blood Disorders Center, Children’s Healthcare of Atlanta, Atlanta, GA, United States; ^2^ Department of Pediatrics, Emory University School of Medicine, Atlanta, GA, United States

**Keywords:** kynurenine, PGE2, heme oxygenase-1, hypoxia-inducible factor 1 α, indoleamine 2,3-dioxygenase, graft versus host disease, mesenchymal stromal cell, aerobic glycolysis

## Abstract

Mesenchymal stromal cells (MSCs) are spindle-shaped, plastic-adherent cells *in vitro* with potent immunosuppressive activity both *in vitro* and *in vivo*. MSCs have been employed as a cellular immunotherapy in diverse preclinical models and clinical trials, but most commonly as agents for the prophylaxis or therapy of graft versus host disease after hematopoietic cell transplantation. In addition to the oft studied secreted cytokines, several metabolic pathways intrinsic to MSCs, notably indoleamine 2,3-dioxygenase, prostaglandin E2, hypoxia-inducible factor 1 α, heme oxygenase-1, as well as energy-generating metabolism, have been shown to play roles in the immunomodulatory activity of MSCs. In this review, we discuss these key metabolic pathways in MSCs which have been reported to contribute to MSC therapeutic effects in the setting of hematopoietic cell transplantation and graft versus host disease. Understanding the contribution of MSC metabolism to immunomodulatory activity may substantially inform the development of future clinical applications of MSCs.

## Introduction

First identified in bone marrow in 1968 (1, 2), mesenchymal stromal cells (MSCs) are spindle-shaped, adherent in cell culture conditions, and widely investigated for their immunoregulatory properties and ability to contribute to tissue regeneration (3, 4). MSCs have been identified in a variety of tissue sources including bone marrow, adipose tissue, amniotic membrane and fluid, placental and fetal tissues, umbilical cord tissues, endometrium, blood, and synovial fluid (3). MSCs are present in relatively low numbers in any given tissue, and thus, prior to research or clinical use, are isolated and expanded *ex vivo* in cell culture media (3). Since MSCs may display several morphological and physiological characteristics in culture (3, 5), the minimum necessary criteria for MSC definition have been outlined by the International Society for Cellular Therapy (6, 7).

Indeed, MSCs are known to differentiate into chondrocytes, adipocytes, and osteocytes *in vitro*, suggesting that perhaps MSCs perform stem-like functions (8–12). Although tissue repair and regeneration has been reported after intravenous infusion of MSCs in a variety of disease settings (e.g., and late-onset hemorrhagic cystitis) (13–17), there are currently no *in vivo* data demonstrating that MSCs are true stem cells, differentiating to resident cells; however, it is possible that MSCs indirectly mediate endogenous tissue regeneration and repair mechanisms, perhaps by secreting soluble factors, *via* paracrine mechanisms or metabolic activity (18).

MSCs have also been shown to modulate adaptive and innate immunity in vitro and in vivo, usually after cytokine activation (e.g., IFN-γ) (4). MSCs may suppress both T and B cell proliferation as well as T cell effector activity (19–23), and MSCs are reported to inhibit proliferation by arresting T cells in the G0/G1 phases of the cell cycle or by promoting lymphocyte apoptotic pathways (24–28). Mechanistically, MSCs likely contribute to immunomodulation through cell-to-cell contact or paracrine and metabolic mechanisms (e.g., TGF-β, hepatocyte growth factor, prostaglandin E2; PGE2, and indoleamine 2,3-dioxygenase; IDO pathways), as immunosuppression has been reported in co-cultures and when cells are separated by a Transwell (26, 27, 29–32). Regarding the innate immune system, MSCs activated by monocytes or cytokines signal macrophages to promote pro- or anti-inflammatory pathways, by inducing the polarization of M2 macrophages (30, 32, 33). Moreover, inactive or apoptotic MSCs, those engulfed by phagocytic cells, or suppressed by host cytotoxic cells contribute to immunosuppression in in vitro and in vivo (34–36).

On account of this immunosuppressive activity, MSCs have been identified as promising candidates for immunosuppressive cell therapies and have been especially studied in the context of treating and preventing acute graft versus host disease (aGVHD) during hematologic cell transplantation (HCT), which occurs when donor immune cells attack recipient tissue (usually liver, gut, and skin) (37, 38). MSC-based cell products have been approved or conditionally approved for the treatment and prophylaxis of aGVHD in pediatric patients in Japan (TEMCELL), Canada, and New Zealand (Prochymal) (39). Recently, Mesoblast conducted a phase III clinical trial using a donor-derived bone marrow MSC cell therapy (RYONCIL™) to treat pediatric steroid refractory aGVHD (40). Notwithstanding these approved and pre-approved MSC-based cell products for treatment and prophylaxis of GVHD, many clinical trials have generated mixed results (40–44).

**Figure 1:**
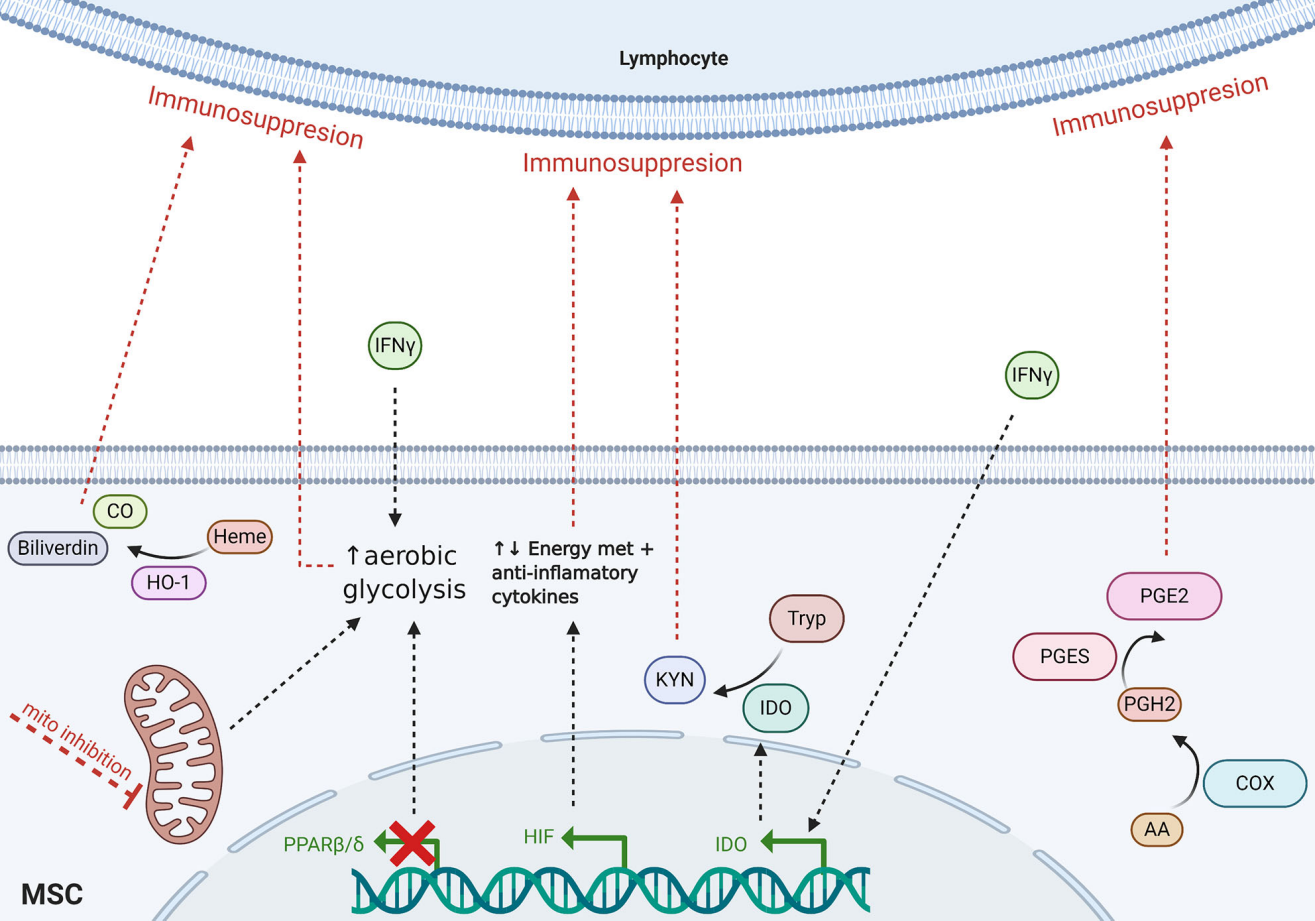
Schematic of proposed MSC-mediated metabolic immunomodulatory mechanisms reviewed herein. Dotted lines indicate incompletely understood mechanisms. Tryp, tryptophan; Kyn, kynurenines; CO, carbon monoxide; AA, arachidonic acid; mito, mitochondria; met, metabolism.

Although cell contact-dependent and secretory mechanisms have been established as the primary immunoregulatory modes of action of MSCs, recently, metabolic stress and activity have been shown to be involved in MSC immunomodulatory functions. Indeed, a great many metabolic pathways are known known to be involved in MSC physiologic mechanisms. However, the IDO, PGE2, hypoxia-inducible factor 1 α (HIF1α), heme oxygenase-1 (HO-1), and energy metabolic pathways have been especially implicated in the literature to play key roles in the immunosuppressive activity of MSCs (**Figure 1**). Herein, we review recent and notable scientific advances that indicate how the aforementioned metabolic pathways endogenous to MSCs, among others, may contribute to immunomodulation in the context of HCT and GVHD.

## IDO: IFN-γ and the Kynurenine Pathway

The metabolic activity of IDO seemingly plays a key role in the immunosuppressive activity of MSCs on T cells and other lymphocytes (45, 46). Naïve MSCs do not normally synthesize IDO; however, after cytokine activation (usually by IFN-γ or TNF-α), MSCs express high levels of IDO (22, 47) The enzymatic activity of IDO acts on the kynurenine pathway of tryptophan metabolism. An essential amino acid, tryptophan is recruited by the cell for protein synthesis, or may be metabolized in the serotonin or tryptamine pathways (48, 49). After induction of IDO expression, the kynurenine pathway is activated and L-tryptophan is metabolically converted into kynurenine, which may be further metabolized into biologically-active kynurenine derivatives including kynurenic acid, 3-hydroxyanthranilic acid, picolinic acid, quinolinic acid (48, 49). Notably, quinolinic acid and 3-hydroxyanthranilic acid are known to target lymphocytes and contribute to suppression of T cell proliferation (50, 51), and kynurenic acid may also modulate the immune system by agonizing aryl hydrocarbon receptor, G-protein-coupled receptor 35, and promoting anti-inflammatory cytokines (e.g., TNF-α, IL6, IL1β, and IL10) (52). Moreover, the addition of tryptophan significantly restores allogeneic T-cell proliferation (53), while adding kynurenine suppresses allogeneic T-cell proliferation (46). Although IDO-mediated catabolism of tryptophan contributes to the MSC-induced immunosuppression, additional investigation is needed in order to uncover the mechanism of action, especially *in vivo*.

## IDO: Immunomodulation and GVHD

It is known that MSCs elicit immunosuppressive effects when first primed with IFN-γ or a combination of IFN-γ with TNF-α, IL-1α or IL-1β (22, 47). Given that the IDO pathway is significantly upregulated with IFN-γ treatment, IDO expression and tryptophan metabolism has been implicated in suppressing T cells and controlling GVHD (54). Kim et al. recently demonstrated that compared to naïve MSCs, human MSCs primed with IFN-γ significantly upregulated IDO expression, increased immunosuppressive activity *in vitro*, and reduced GVHD symptoms and mortality a NOD-SCID PBMC-transplanted mouse model (55). In addition, the investigators showed that downregulating IDO in IFN-γ-primed MSCs decreased this activity, and IDO expression was driven by the JAK/STAT1 signaling pathway. In another recent study, human gingival MSCs stimulated were reported to inhibit T cell and PBMC proliferation *in vitro* and improve survival in a xenogenic GVHD model in the NOD/SCID mice *via* a combination of CD39, CD73, adenosine, and IDO signals (56).

Inhibition of T cell proliferation by IFN-γ-licensed MSCs is widely believed to be IDO-dependent. However, there is additional evidence that IDO metabolism and signaling may not be involved with MSC activity on effector T cell effector functions (e.g., cytokine production). Chinnadurai et al. have shown, for example, that IFN-γ-primed MSCs inhibit T cell (Th1) effector production of IFN-γ, TNF- α, and IL-2 independent of IDO. Inhibition of T cell effector function was instead mediated by B7H1 and B7DC/PD1 pathways (23). In an analysis of a MSC-based off-the-shelf cell product, intravenous infusions of Cymerus™ MSCs (Cynata Therapeutics) ameliorated disease and prolonged survival in a humanized GVHD mouse model after treating cells with IFN-γ (57). Although activating Cymerus™ MSCs with IFN-γ increased IDO expression 5-fold after a 48 h incubation, upregulation of the immune checkpoint inhibitor PD-L1, which contributes to the PD1-PDL1 signaling axis was also observed (26, 58). Therefore, while IDO-meditated metabolic activity plays a role in the immunomodulatory properties of IFN-γ-primed MSCs, other factors such as PD1 signaling and IDO-independent T cell effector activity seem to also be involved in immunosuppression induced by MSCs. Moreover, the relative contributions of each mechanism have yet to be determined.

Despite the potent immunosuppressive activity observed in IFN-γ-primed MSCs, largely due to the IDO pathway, no clinical trials to date have employed IFN-γ-primed MSCs for the treatment and prophylaxis of GVHD. However, Horwitz and colleagues have recently registered a phase I clinical trial with the NIH aimed at using IFN-γ-primed MSCs as prophylaxis for aGVHD after patients with hematologic malignancies and myelodysplasia have received HCT (NCT04328714).

## Prostaglandin E2

PGE2 metabolic activity has been implicated in MSC-based immunosuppressive activity. PGE2 is an arachidonic acid derivative synthesized by cyclooxygenases COX1, COX2, and prostaglandin synthetase (59). MSC secretion of PGE2 correlates with suppression of lymphocyte proliferation (60), and PGE2 is known to promote induction of immunosuppressive interleukins (IL4, IL10, and IL6), proliferation and cytotoxicity of natural killer cells, and differentiation of Treg cells, and suppress differentiation of dendritic cells and naive T cells to Th17 cells (61–64),. Additionally, IL6 may contribute to PGE2-mediated immunomodulation in MSCs by positively regulating the COX2 function and synthesis of PGE2 (65, 66). IL6-dependent PGE2 has also been shown to promote immunosuppression *via* changing Th1 and Th2 ratios, inhibiting maturation of dendritic cells and stimulating of Treg cells (67, 68). Thus, synthesis and secretion of PGE2 contributes to MSC immunomodulation and have immunosuppressive potential in the context of GVHD.

*In vivo*, MSC infusions significantly increase secretion of PGE2 both before and after the onset of GVHD (69). Auletta et al. reported that of indomethacin (IM), a COX inhibitor which decrease PGE2 synthesis, and direct pharmacologic inhibition of PGE2-EP receptor interaction reversed T cell suppression induced my BM MSCs *in vitro* (70). In an allogeneic BMT mouse model, the investigators show that the survival advantage of animals treated with intravenous BM MSC injections was also reversed with a 7-day dose of IM. Similar results have been reported with MSCs isolated from other tissue sources, such as umbilical cord tissue (71). More recently, Kim et al. showed that treating BM MSCs with IM or downregulating expression of prostaglandin E synthetase (PGES) *via* siRNA reduced proliferation of human PBMCs, and PGES knock down MSCs were unable to reduce mortality in mice with GVHD (72). These studies present evidence that PGE2 may be a key effector of immunosuppression in GVHD and HCT clinical settings.

## Heme-Oxygenase-1

Heme oxygenase intracellularly metabolizes heme to biliverdin, CO, and free divalent iron, and HO-1 is reported to have anti-inflammatory and immunosuppressive properties (73–75). Chabannes et al. (76). were the first to demonstrate that HO-1 may play a role in MSC-mediated immunosuppression, and report a reversal of PBMC suppression after adding the HO-1-specific inhibiter tin protoporphyrin (SnPP) (76). Reduced T cell suppression *in vitro* and improved survival *in vivo* was also observed after inhibiting rat MSC HO-1 in combination with nitric oxide synthase (NOS). Human MSCs displayed similar results *in vitro*, but were not tested *in vivo*. Interestingly, the nitric oxide synthesizing pathway, which, like the IDO pathway, may also be stimulated in MSCs by IFN-γ, is implicated in rodent rather than human systems (77–79). Infusion of murine MSCs transduced with murine HO-1 have also been shown to increase the number of Treg cells in spleen and lymph nodes, and significantly reduce severity of clinical aGVHD in mice (80).

In contrast, Galipeau and colleagues have reported that human BM MSCs express low levels of HO-1 both before and after priming with IFN-γ, TNF-α, and/or TGF-β, and MSCs treated with SnPP had no effect on T cell suppression, possibly due to the notion that the IDO pathway may require heme as a cofactor (81). Therefore, the role of HO-1 in MSC-mediated immunomodulation, notably in the context of suppressing the immune system, is inconsistent in the literature. It is possible that this inconsistency may be an *in vitro* artifact, or a result of mixing experimental approaches. For example, studies described herein that reported an effect of HO-1 on MSC-mediated immune suppression, largely studied animal MSCs, while Galipeau et al. investigated humans MSCs. In any case, additional investigation is required in order to understand the role of HO-1 in MSC-mediated immunomodulation, especially *in vivo*.

## Hypoxia-Inducible Factor 1 α

*In vivo*, MSCs are thought to be located in perivascular niches under relatively hypoxic conditions, where oxygen tension is low. Hypoxia plays a crucial role in maintaining homeostasis throughout the body from early stages of embryonic development, and the metabolic regulatory mechanisms of hypoxia are largely driven by oxygen-sensitive transcription factors, including hypoxia-inducible factor 1 (HIF-1) (82). HIF-1 is a heterodimer consisting of an oxygen-regulated α-subunit and a constitutively expressed β-subunit. Under hypoxic conditions, hydroxylation of HIF-1 α by prolyl hydroxylase is suppressed, leading to the accumulation and nuclear translocation of HIF-1 α (83, 84). Activation of HIF-1 α has been shown to regulate transcription of genes necessary for carbohydrate, fatty acid, and other metabolic pathways involved with energy production (85). Metabolic activity and regulation of HIF-1 α in MSCs has been implicated in MSC differentiation potential (86–89), migration and chemotactic localization (90), the inflammatory response (91), tissue repair, and angiogenesis (92–94)

Hypoxia and HIF-1 α metabolism in MSCs may also play a role in immunomodulation. In response to hypoxia, it has been shown that MSCs produce an increased level of anti-inflammatory cytokines (e.g., IL-10), decreased pro- inflammatory cytokines (e.g., TNF α), and demonstrate enhanced suppression of PBMCs (95–97). Recently, Kim and colleagues have shown that human MSCs expanded under hypoxia, promoted T cell suppression, and when IV-administered to a humanized mouse GVHD model, improved survival and reduced symptoms of GVHD were also observed (97). These data support the notion that hypoxia priming or increased expression HIF-1 α in MSCs could be a viable strategy to promote donor and host immunomodulation and reduce GVHD during HCT. However, hypoxia- and HIF-1 α-mediated immunoregulation by MSCs is a relatively new avenue of research, and this transcription factor regulates many metabolically active genes. Thus, additional studies aimed at uncovering a mechanism of action are needed.

## Energy Metabolism

Energy-generating metabolic pathways (i.e., lipid and carbohydrate metabolism) have also been implicated in MSC-mediated immunomodulation. Contreras-Lopez et al. recently demonstrated that metabolism of peroxisome proliferator-activated receptor (PPAR) β/δ, which plays and key role in lipid and glucose metabolism and homeostasis, may be important for MSC immunomodulation of T cells (98). The investigators reported that PPARβ/δ knock out MSCs had enhanced suppression of Th1 and Th17 proliferation *via* enhancement of glycolysis, and inhibition of mitochondrial generation of ATP promoted aerobic glycolysis in WT MSCs and consequentially improved immunosuppressive activity. Similarly, Lui et al. recently demonstrated that priming MSCs with IFN-γ induces a metabolic switch towards aerobic glycolysis and strengthens T cell suppression (99). Moreover, oxidative glucose and lipid metabolism have been shown to contribute only 3% of ATP production in MSCs, while glycolysis generates 97% of cellular ATP (100). The studies together suggest that regulation of energy metabolism in MSCs, notably by reprogramming a switch from mitochondrial activity towards glycolysis, promotes immunosuppression.

## Conclusion

MSCs contribute to adaptive and innate immunomodulation through cell-to-cell contact, secretory and paracrine signaling mechanisms, as well as intracellular metabolic pathways. IFN-γ, IDO and kynurenine, PGE2, HIF1α, HO-1, as well as energy-generating metabolic pathways have been implicated in MSC-mediated immunosuppression. Some studies have reported conflicting results, particularly regarding specific mechanisms of action and downstream targets. Moreover, the role of HO-1 in immunomodulation by MSCs remains an open question. IDO-kynurenine metabolism presents one of the most compelling mechanisms by which MSCs suppress the immune system. However, given that IDO is only expressed after MSCs are primed with IFN-γ or other cytokine combinations, which may regulate expression and activation of other factors (e.g., PDL-1), IDO may be only one of many contributors of MSC-based immunoregulation. Interestingly, aerobic glycolytic pathways, rather than oxidation of energy-generating substrates *via* mitochondria, have recently been hypothesized to play a key role in MSC immunomodulation, adding to the studies that promote the importance of IDO and kynurenine metabolism *via* IFN-γ licensing. Understanding how MSC metabolism modulates immune cell activity may have significant applications in the development of MSC-based therapeutics, especially in the context of HCT and aGVHD.

## Author Contributions

AJB and EMH conceived and designed the review. AJB wrote the original manuscript draft. EMH, EMF, and AJB edited and approved the final version of the manuscript. All authors contributed to the article and approved the submitted version.

## Funding

This work was supported by the National Institutes of Health (grant R56HL147867) and Aflac Cancer & Blood Disorders Center.

## Conflict of Interest

The authors declare that the research was conducted in the absence of any commercial or financial relationships that could be construed as a potential conflict of interest.
